# SUPPORTING THE UPTAKE OF PERSON-CENTERED COMMUNICATION STRATEGIES IN NURSING HOMES USING THE PRISM

**DOI:** 10.1093/geroni/igae098.0158

**Published:** 2024-12-31

**Authors:** Natalie Douglas

**Affiliations:** University of Louisiana at Lafayette, Lafayette, Louisiana, United States

## Abstract

The implementation of quality, person-centered care into nursing home (NH) settings is challenging due to the complexity of NH organizational structures, nationwide staffing shortages, and significant federal regulations. We used the Practical, Robust Implementation and Sustainability (PRISM) Model to assess key contextual factors that may influence the implementation of Dementia Collaborative Coaching, an evidence-informed communication coaching program designed to decrease negative, responsive behaviors and improve quality of life for people living with dementia in NHs. Employees (n=27) from three urban NHs including corporate leaders, managers, and direct care providers across a variety of disciplines engaged in n=5 focus group sessions to deepen understanding of barriers and facilitators to the implementation of Dementia Collaborative Coaching. Participants identified barriers to Dementia Collaborative Coaching implementation including the need to improve dementia care overall, inadequate leadership support, staffing concerns, and misinterpretation of NH federal regulations. Facilitators included enthusiasm for the program as well an agreed upon need for an NH-wide implementation and sustainability plan. Outcomes most relevant to the participants included decreased behavioral incidents and falls as well as increased engagement in both leisure and activities of daily living. Pre-implementation guided by the PRISM proved to be a critical step in maximizing the success of implementation of this intervention and developing relationships with key players in the NH. During this process, site-specific system and clinician level factors will inform future Dementia Collaborative Coaching implementation efforts which may also contribute to the implementation of other quality interventions for NH residents.

